# Impact of Yttrium Oxide on the Synthesis and Sintering Properties of Cordierite–Mullite Composite Ceramics

**DOI:** 10.3390/ma18030687

**Published:** 2025-02-04

**Authors:** Hui Zhang, Lu Feng, Weibo Mao, Quanming Liu, Liang Zhao, Hong Zhang

**Affiliations:** 1 College of Civil Engineering, Xi’an University of Architecture and Technology Hua Qing College, Xi’an 710043, China; jdfenglu@126.com (L.F.); wweibomao@163.com (W.M.); zhanghong198806@163.com (H.Z.); 2 Gas Turbine Technology Department, Xi’an Thermal Power Research Institute Co., Ltd., Xi’an 710032, China; liuquanming1988@126.com; 3 School of Civil Engineering and Architecture, Yangtze Normal University, Chongqing 408100, China; zhaoliang@yznu.edu.cn

**Keywords:** cordierite–mullite, sintering aid, yttrium oxide, thermal shock stability, microstructure

## Abstract

To enhance the mechanical properties and high-temperature performance of cordierite–mullite composite ceramics, yttrium oxide (Y_2_O_3_), a rare earth metal oxide, was employed as a sintering aid to fabricate these composites via in situ synthesis and non-pressure sintering. This study systematically investigated the formation mechanisms of the cordierite and mullite phases and examined the effects of yttrium oxide on the densification behavior, mechanical properties, volumetric stability, and thermal shock resistance. The results indicate that incorporating yttrium oxide (1.5–6.0 wt%) not only promoted the formation of the cordierite phase but also refined the microstructure and enhanced the thermal shock stability at a sintering temperature of 1350 °C. An optimal addition of 3 wt% yttrium oxide ensures that the primary phases are cordierite and mullite, with a microstructure characterized by uniformly distributed micropores, hexagonal short-columnar cordierite, and interlocking rod-like mullite, thereby significantly improving both the mechanical properties and thermal shock stability. Specifically, the room-temperature compressive strength increased by 121%, the flexural strength increased by 177%, and, after three thermal shock cycles at 1100 °C, the retention rates for compressive and flexural strengths were 87.66% and 71.01%, respectively. This research provides a critical foundation for enhancing the mechanical properties and high-temperature service performance of cordierite–mullite saggers used in lithium battery cathode materials.

## 1. Introduction

The rapid development of the global economy has led to an increasing demand for traditional energy sources, resulting in severe environmental issues and energy crises. In response, researchers in China and other countries have been actively developing green, low-carbon, and environmentally friendly energy materials. Lithium-ion batteries, with their high energy storage density, specific heat capacity, thermal stability, long service life, and low manufacturing costs, have become integral to the advancement of new energy technologies. They have been widely used in sectors such as digital electronics, hybrid electric vehicles, and battery electric vehicles [1,2,3]. Notably, China’s “14th Five-Year Plan” designates the “New Energy Industry” as a key focus area for driving the rapid expansion of the new energy vehicle market. As the “core” component of new energy vehicles, lithium batteries are experiencing rising demand, which, in turn, increases the need for saggars to be used in the sintering of lithium battery cathode materials [4,5,6]. However, these saggars are prone to erosion by lithium oxide and thermal shock damage during operation, reducing the production efficiency of cathode materials. Therefore, advancing technological innovation and exploring high-performance saggars for sintering lithium battery cathode materials is of practical significance in boosting the growth of the new energy industry.

The primary types of saggars include oxide-based and non-oxide-based types [7,8,9], namely alumina–silica saggars (mullite-based), alumina–silica–magnesia saggars (cordierite-based), and carbide and nitride saggars, such as silicon carbide and silicon nitride. However, single-component saggars often fail to meet the requirements of current technological advancements. Combining different components can synergistically complement their strengths and weaknesses, resulting in composite saggars with superior performance [10,11,12]. For instance, cordierite–mullite composite ceramics [13,14,15] can retain high thermal stability and mechanical strength from mullite while maintaining the low thermal expansion coefficient and excellent thermal shock stability of cordierite. These properties make such composites particularly suitable for preparing saggars used in the sintering of lithium battery cathode materials.

To date, scholars have investigated the variation patterns of the properties of cordierite–mullite composite ceramics from multiple perspectives, including the raw material types, preparation processes, and admixture types. This research has established a critical theoretical foundation for enhancing the performance optimization and extending the service life of cordierite–mullite saggers. Sun et al. [16] investigated the effect of calcium titanate aluminate (CTA), a metallurgical solid waste, on a mixture of cordierite, mullite, alumina, white clay, and talc. Their results revealed that incorporating 6 wt% CTA facilitated the removal of pores and a densification of the sintering process, owing to the presence of an appropriate amount of liquid phase. This optimization improved the high-temperature stability and thermal shock resistance of the cordierite–mullite saggars. Hu et al. [17] utilized fly ash, acid-treated talc and acid–alkali-treated talc as primary raw materials to fabricate composite ceramics. When sintered at 1370 °C for 2 h with a cordierite-to-mullite ratio of 50:50, the resulting ceramics met the basic property requirements of cordierite–mullite saggars. Similarly, Xie et al. [18] employed cordierite and mullite as the main components and alumina, kaolin, and talc as auxiliaries to prepare saggars at 1350 °C. The obtained samples achieved a dense structure, uniform pore distribution, high mechanical strength, and excellent thermal shock stability. The fusion of cordierite and mullite particles with the matrix further enhanced their erosion resistance. Kakroudi et al. [19] utilized clay, talc, and andalusite as the primary raw materials, incorporating a specific proportion of silicon carbide. At 1375 °C for 1 h, when the silicon carbide content was 5wt%, the silicon carbide particles facilitated the transformation of andalusite to mullite, altered the direction of crack propagation, and inhibited crack propagation. The mechanical properties and thermal shock stability were characterized.

While the aforementioned research has enhanced the fundamental properties of cordierite–mullite composite ceramics to some extent, several challenges remain. These include high sintering temperatures, reliance on pre-synthesized raw materials, and low residual strength after thermal shock. These issues result in a complex preparation process, high energy consumption, and elevated production costs, which are inconsistent with the principles of “energy conservation and emission reduction”. Consequently, numerous scholars have introduced sintering aids to promote low-temperature densification and the sintering of ceramic materials through liquid-phase sintering. Commonly used sintering aids include alkali metal/alkaline earth metal oxides, transition metal oxides, and rare earth metal oxides. Studies have demonstrated that rare earth metal oxides [20,21] (such as cerium oxide, lanthanum oxide, yttrium oxide, neodymium oxide, and samarium oxide) can significantly enhance the densification and sintering processes of ceramic materials, thereby markedly improving their mechanical properties and high-temperature performances. Hu et al. [22] investigated the impact of yttrium oxide on the properties of cordierite-based composite ceramics when used as a sintering agent. They reported that adding 7wt% yttrium oxide facilitated the fabrication of cordierite-lithium-zircon composite ceramics with high densification and an excellent thermal shock stability, thereby establishing a significant foundation for the application of cordierite-based composites in solar power generation ceramic pipelines. Avcioglu and Artir [23] fabricated cordierite–mullite–zircon composite ceramics using yttrium oxide as a sintering agent, which facilitated the densification and sintering process, increased the flexural strength by sixfold, and significantly enhanced the neutron and gamma-ray shielding capabilities, thus expanding the potential applications of these ceramics in radiation environments. Despite these advancements, research on the synthesis mechanisms and performance improvements of yttrium oxide composite ceramics remains limited. Non-pressure sintering and in situ synthesis methods have been employed to reduce the energy consumption and production costs associated with cordierite–mullite composite ceramics. This study examined the effects of yttrium oxide on the densification, sintering behavior, mechanical properties, and thermal shock stability of these composite ceramics to provide a theoretical basis for enhancing their mechanical properties and high-temperature performance.

## 2. Experiments

### 2.1. Raw Materials

The materials used in this study included calcined kaolin (industrial grade, particle size 0.044 mm, Henan Borun New Materials, Zhengzhou, China), sintered magnesia (industrial grade, particle size 0.074 mm, Sinosteel Luoyang Institute of Refractories Research Co., Ltd., Luoyang, China), alumina powder (analytical grade, particle size 0.001 mm, Nano New Materials Technology Co., Ltd., Shanghai, China), rare earth metal oxide yttrium oxide (Y₂O₃, analytical grade, particle size 0.044 mm, sintering aid, Shanghai Macklin Biochemical Technology Co., Ltd., Shanghai, China), and polyvinyl alcohol (PVA, analytical grade, particle size 0.044 mm, binder for compression molding, Shanghai Chenqi Chemical Technology Co., Ltd., Shanghai, China). Additionally, deionized water, prepared in the laboratory, was used as the solvent for the PVA solution. The chemical compositions of the materials were analyzed using a Rigaku ZSX Primus III+ X-ray fluorescence spectrometer (Rigaku Research Institute, Fuso Industrial Co., Ltd., Tokyo, Japan), with the results being listed in Table 1.

### 2.2. Preparation of Samples

**Figure 1:**
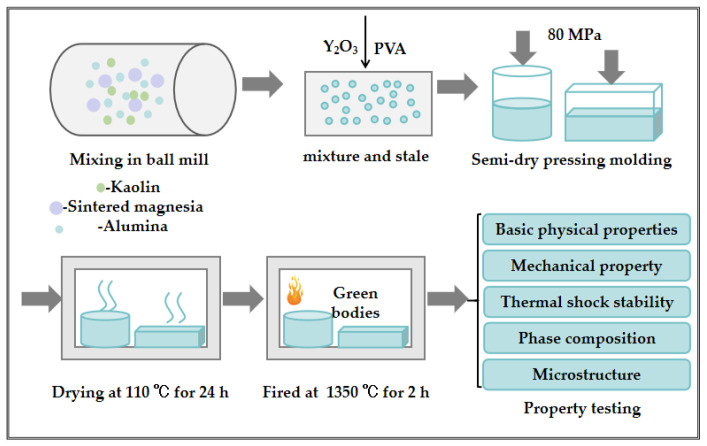
Preparation process of cordierite–mullite composite ceramics.

The cordierite–mullite composite ceramics were prepared following the process shown in Figure 1. The detailed preparation procedure is outlined as follows.

#### 2.2.1. Formulation Design

According to the phase diagram of the MgO-Al_2_O_3_-SiO_2_ ternary system, positioning the composition point along the M₂A₂S₅-A₃S₂ (where M represents MgO, A represents Al_2_O_3_, and S represents SiO_2_, while M_2_A_2_S_5_ denotes cordierite and A_3_S_2_ signifies mullit) join line prevents the formation of excessive liquid phases during the service time of the sample and the influences its high-temperature performance [24]. In this study, the sample formulation was calculated based on the theoretical chemical compositions of cordierite and mullite, with a mass ratio of 70:30 for cordierite to mullite. The purpose was to evaluate the influence of adding yttrium oxide (sintering aid) to the properties of the samples. The formulation design is displayed in Table 2. When the addition of yttrium oxide increased from 0 wt% to 6.0 wt%, the sample designations were C-M-Y0, C-M-Y1, C-M-Y2, C-M-Y3, and C-M-Y4, respectively.

#### 2.2.2. Mixing and Molding

The raw materials were thoroughly mixed in a rotary ball mill using alumina balls as the grinding media. The ball-to-material ratio was set at 1:2, with a rotation speed of 80 rpm and a milling time of 4 h. Subsequently, a 3 wt% PVA solution was added as a binder. The mixture was kneaded uniformly with a ceramic roller and conditioned in a sealed bag for a 12 h conditioning process to ensure even binder distribution within the ceramic powders. The prepared ceramic powders were then molded into cylindrical and rectangular green bodies with dimensions of φ30 mm × h 30 mm (the internal diameter of the mold measures is 30 mm and its depth is 80 mm) and 15 mm × 15 mm × 100 mm (the internal dimensions of the mold are as follows: length 100 mm, width 15 mm, and depth 30mm), respectively, under an uniaxial pressure of 80 MPa using a TYE-300B compression-testing machine provided by Wuxi Jianyi Experiment Instrument Co., Ltd. (Wuxi, China).

#### 2.2.3. Drying and Sintering

The molded green bodies were dried at 110 °C for 24 h in a 101-3 electrothermal blowing dry box (Beijing Kewei Yongxing Instrument Co., Ltd., Beijing, China) to eliminate mechanically bound water. Following drying, the ceramic green bodies were thermally treated at 1350 °C (the theoretical synthesis temperature for cordierite is approximately 1400 °C) in an SGM M25/16E AI box-type resistance furnace (Luoyang Sigma High-Temperature Electric Furnace Co., Ltd., Luoyang, China) with a holding time of 2 h at the peak sintering temperature.

### 2.3. Performance and Characterization Techniques

The bulk density, apparent porosity, and water absorption of the sintered samples were tested according to the standard GB/T 2997-2015 [25], employing vacuum impregnation and drainage methods. The instruments used a ZXE-Z rotary-vane vacuum pump (Beijing Zhongxing Weiye Instrument Co., Ltd., Beijing, China) and an XQ-01 measurement instrument for apparent porosity and bulk density (Beijing Jinyang Wanda Technology Co., Ltd., Beijing, China). Compressive strength testing of the sintered samples at room temperature adhered to the standard GB/T 5072-2008 [26], performed using a TYE-300B compression-testing machine (Wuxi Jianyi Experiment Instrument Co., Ltd., Wuxi, China). Flexural strength was assessed in accordance with the GB/T 4741-1999 standard [27] using the NO3074 anti-fracture-testing machine made in Japan. The thermal shock stability of the samples was evaluated using an SX3 muffle furnace from Hangzhou Zhuochi Instrument Co., Ltd. (Hangzhou, China). The testing conditions involved heating the samples to 1100 °C and maintaining this temperature for approximately 20 min. The samples were then rapidly immersed in flowing cold water for about 10 min, followed by a 5-minute air-cooling phase. This cycle was repeated three times, and the strength retention of the flexural strength and the compressive strength of the samples was measured to characterize their thermal shock stability. Additionally, the linear dimensional changes upon re-sintering were measured following the GB/T 5988-2022 standard [28]. The dimensional change rate before and after sintering at 1100 °C was determined to evaluate the volume stability of the samples under high-temperature service conditions.

Following the strength tests, the samples were ground to particle sizes not exceeding 74 μm. The phase composition of the samples was analyzed using a Bruker D8 Advance X-ray Diffractometer (Bruker Corporation, Saarbrucken, Germany) with Jade 6.5 software. The testing parameters included a copper target, a scanning speed of 10 °/min, and a scanning range of 5−90°, and the testing step size was 0.02 degrees. Finally, samples with dimensions smaller than 10 mm were selected for cross-sectional microstructure characterization and energy spectrum analysis using a ZEISS Sigma 300 scanning electron microscope (Carl Zeiss AG, Oberkochen, Germany) and a British OXFORD Xplore energy dispersive spectrometer (EDS, Oxford Instruments Technology Co., Ltd., Oxford, UK). The samples were mounted on conductive adhesive and gold coated for approximately 45 s using a Quorum SC7620 sputter coater (Quorum Corporation, Ringmer, Britain). Subsequently, the scanning electron microscope was used to obtain the cross-sectional microstructure and energy dispersive spectroscopy (EDS) of the samples. The accelerating voltage was set to 3 kV for topographical imaging, while it was adjusted to 15 kV for EDS point scans. Secondary electron images were acquired using the SE2 detector.

## 3. Results and Discussion

### 3.1. Thermodynamic Calculation of Cordierite–Mullite Composite Ceramics

**Figure 2:**
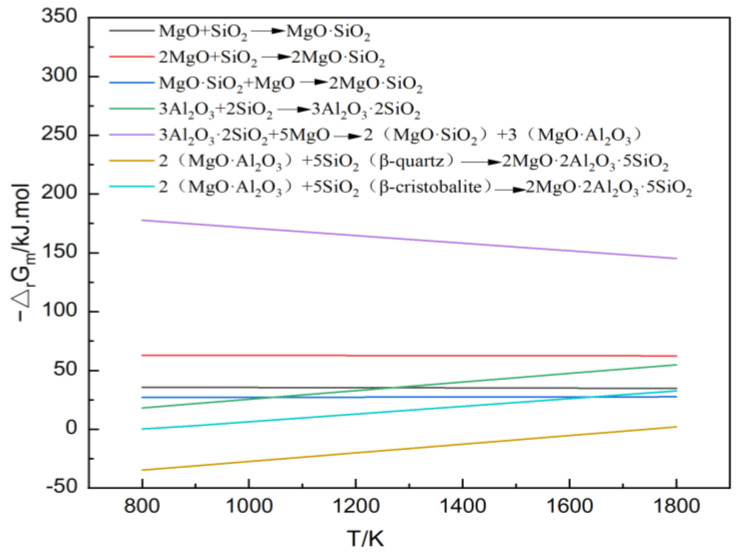
Thermodynamic calculation of the MgO-Al_2_O_3_-SiO_2_ ternary system.

The sintering temperature has a significantly greater impact on the free energy of a reaction compared to the reactant concentration and partial pressure. Therefore, the relationship between temperature and the free energy of a standard reaction can be visually represented through thermodynamic calculations. In this study, the cordierite and mullite phases were formed via in situ solid-state reactions between alumina (Al₂O₃), silica (SiO₂), and magnesia (MgO) in the raw materials. The theoretical Gibbs energies of each reaction were calculated over the temperature range of 800K to 1800K. The calculated results were then plotted as a linear function to illustrate the relationship between the theoretical Gibbs free energies (△_r_G_m_) and temperature (T). The main chemical reactions in the MgO-Al_2_O_3_-SiO_2_ ternary system, along with their thermodynamic calculation results and temperature variation trends, are shown in Figure 2.

As depicted in Figure 2, the sintering temperature had a minimal impact on the Gibbs free energy of the reactions forming enstatite (MgO•SiO₂) and forsterite (2MgO•SiO₂). However, increasing the sintering temperature reduced the formation potentials of mullite (3Al₂O₃•2SiO₂) and spinel (MgO•Al₂O₃) while promoting the synthesis of cordierite (2MgO•2Al₂O₃•5SiO₂). Thermodynamic calculations for the cordierite synthesis (Figure 2) revealed that the theoretical synthesis temperature is approximately 1400 °C. To simplify the preparation process and reduce energy consumption, yttrium oxide was added as a sintering aid. This also allowed for an investigation into its reinforcing mechanism and its influence patterns on the properties of the in situ synthesized cordierite–mullite composite ceramics.

### 3.2. Reinforcing Mechanism of Yttrium Oxide on Cordierite–Mullite Composite Ceramics

When yttrium oxide was added into the MgO-Al₂O₃-SiO₂ ternary system, the relative ionic radius difference (△r) was 22.22% ≤ 30%, satisfying the conditions for limited substitution of Mg^2+^ by Y^3+^ in the cordierite crystal structure [29]. This compatibility arises from the similar ionic radius of Mg^2+^ (r_1_ = 72 pm) and Y³⁺ (r_2_ = 88 pm). Thus, Y^3+^ ions from yttrium oxide partially substituted Mg^2+^ within the cordierite ceramic lattice. Due to the unequal valence of the substituting ions, and as described by the defect equation ($Y_{2}O_{3}\overset{\;MgO\;}{\to }2{Y_{Mg}}^{.}+{V_{\mathrm{Mg}}}^{\prime \prime }+3O_{O};Y_{2}O_{3}\overset{\;MgO\;}{\to }2{Y_{\mathrm{Mg}}}^{.}+{O_{i}}^{\prime \prime }+2O_{O}$), the partial substitution of Mg^2+^ by Y^3+^ led to the formation of a limited solid solution in the lattice accompanied by two forms of point defects (${V_{\mathrm{Mg}}}^{\prime \prime }$ and ${O_{i}}^{\prime \prime }$). These defects promoted the formation of the cordierite and mullite phases.

The addition of yttrium oxide to the sample influenced the synthesis of the cordierite phase in composite ceramics, enabling either a solid-state diffusion process at lower sintering temperatures or a crystallization process facilitated by the liquid phase at higher temperatures [30]. Lattice defects drove ion interactions during the solid-state reaction, while the liquid phase at high temperatures accelerated ion migration. Upon cooling, the glass phase filled gaps between the grains of cordierite and mullite, creating a denser structure. This, in turn, enhanced the load-bearing capacity per unit area of the composite ceramic [31].

The diffusion coefficients of Al^3+^ and Mg^2+^ exceed that of Si^4+^, leading to their migration to the surface of Si^4+^, where they undergo solid-phase reactions to form a cordierite phase at specific temperatures. This process resulted in a cordierite layer of defined thickness on the silica surface, which hindered the continuation of the solid-phase reaction, leaving residual silica and adversely impacting the phase purity and performance of the sample. However, the addition of yttrium oxide to the ceramic raw materials generated a high-temperature liquid phase on the silica surface, notably increasing the diffusion rates of Al^3+^ and Mg^2+^ to the Si^4+^ surface. This promoted the formation of the cordierite and mullite phases, thereby improving the mechanical properties and high-temperature stability of the samples.

### 3.3. Physical Properties, Mechanical Properties, and Thermal Shock Stability

**Figure 3:**
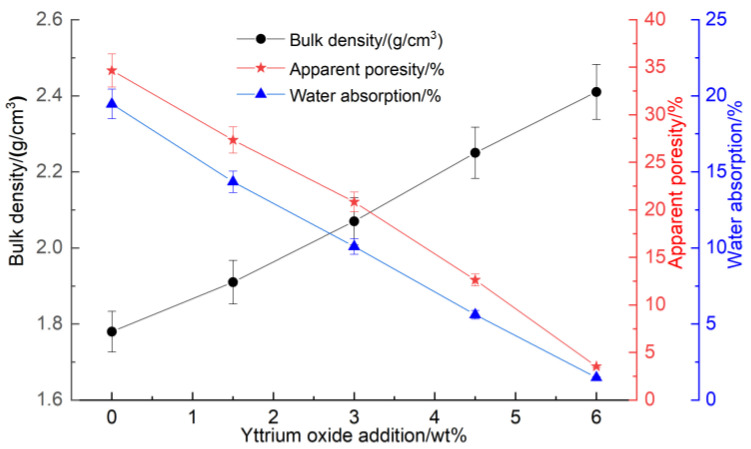
Impact of yttrium oxide addition on the bulk density, apparent porosity, and water absorption of the samples.

Figure 3 presents the impact of the yttrium oxide addition on the apparent porosity, water absorption, and bulk density of the samples at a sintering temperature of 1350 °C.

**Figure 4:**
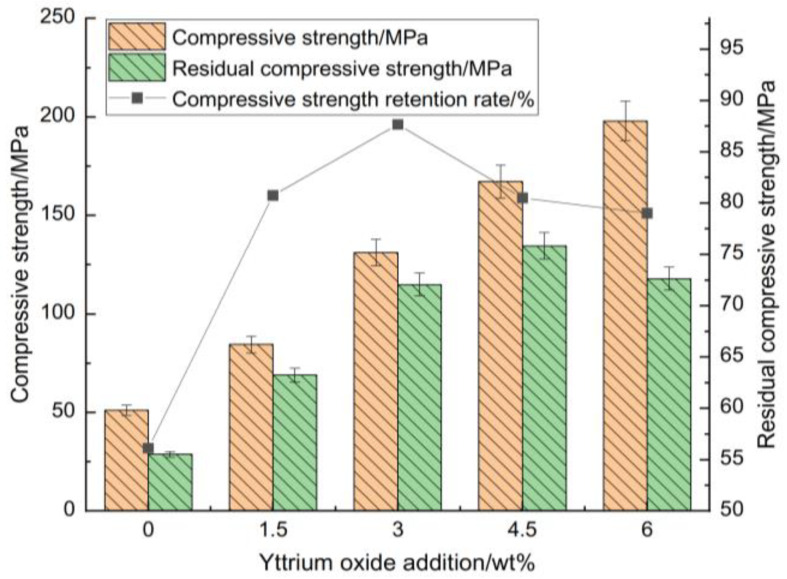
Impact of yttrium oxide addition on the room-temperature compressive strength, thermal shock residual strength, and strength retention rate of the samples.

**Figure 5:**
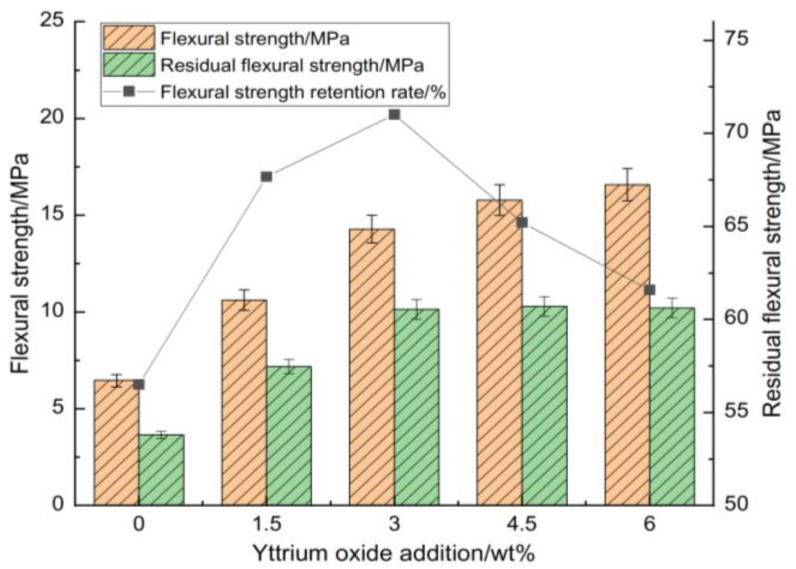
Impact of yttrium oxide addition on the room-temperature flexural strength, thermal shock residual strength, and strength retention rate of the samples.

Figure 4 and Figure 5 highlight its influence on the compressive strength, flexural strength, thermal shock residual strength, and strength retention rate of the samples at room temperature.

At a sintering temperature of 1350 °C for 2 h, the bulk density, compressive strength, and flexural strength increased gradually with the higher yttrium oxide content while the water absorption and apparent porosity decreased. This behavior can be attributed to the ionic substitution of Y^3+^, partially replacing Mg^2+^ in the sample. The unequal substitution resulted in the formation of Y_Mg_^.^ and V_Mg_” defects, causing lattice distortion and enhancing the diffusion rate of ions within the sample. Furthermore, the addition of yttrium oxide reduced the formation temperature of the liquid phase, facilitating its formation on the silica surface at relatively low sintering temperatures. This accelerated the diffusion of Mg^2+^ and Al^3+^ ions to the Si^4+^ surface, promoting the solid-phase reaction among silica, magnesia, and alumina within the sample. Consequently, the densification and sintering processes were accelerated. Furthermore, a higher concentration of yttrium oxide led to the formation of a partial liquid phase that filled the interparticle pores, increasing the sample’s density, which was reflected in the increased compressive and flexural strengths as well as the reduced apparent porosity.

Figure 4 and Figure 5 illustrate that the strength retention rate of the sample after thermal shock increased initially with the addition of yttrium oxide before declining. At a yttrium oxide content of 3 wt%, the residual compressive and flexural strengths of the sample after thermal shock were 114.91 MPa and 10.14 MPa, respectively, corresponding to strength retention rates of 87.66% and 71.01%. This is ascribed to the high purity of the raw materials, which limited the formation of a high-temperature liquid phase during sintering. Meanwhile, the synthesis of cordierite and mullite occurred at elevated temperatures, resulting in a restricted yield, which adversely impacted the mechanical properties and thermal shock stability of the sample. The introduction of yttrium oxide reduced the temperature required for forming the high-temperature liquid phase, facilitating particle rearrangement and enhancing ion migration rate through liquid surface tension and capillary forces. Moreover, an appropriate amount of liquid phase filling the pores between particles promoted the formation of small voids within the sample. These voids enhanced the mechanical properties of the sample and greatly buffered the thermal stress within the sample, contributing to its performance under high temperatures. Conversely, the excessive addition of yttrium oxide during sintering led to the production of a significant high-temperature liquid phase. This liquid phase filled the pores, reducing the energy barrier for crack propagation and adversely impacting the sample’s high-temperature performance. While the mechanical properties showed some improvement, this condition led to significant strength loss following thermal shock.

Table 3 summarizes the linear changes observed in samples re-sintered (LCOR) with varying amounts of yttrium oxide.

Table 3 indicates that the linear change rate upon re-sintering of the samples decreased with the increasing addition of yttrium oxide. In comparison to the blank sample, those containing yttrium oxide exhibited linear change rates upon re-sintering that approached zero. This outcome can be attributed to the absence of significant physical or chemical changes within the samples sintered at 1350 °C, as observed during the linear change rate testing. As a result, the dimensional change rate at this temperature was minimal, suggesting that yttrium oxide addition contributed to enhancing the long-term service stability of cordierite–mullite saggars under high-temperature conditions.

### 3.4. Phase Composition

**Figure 6:**
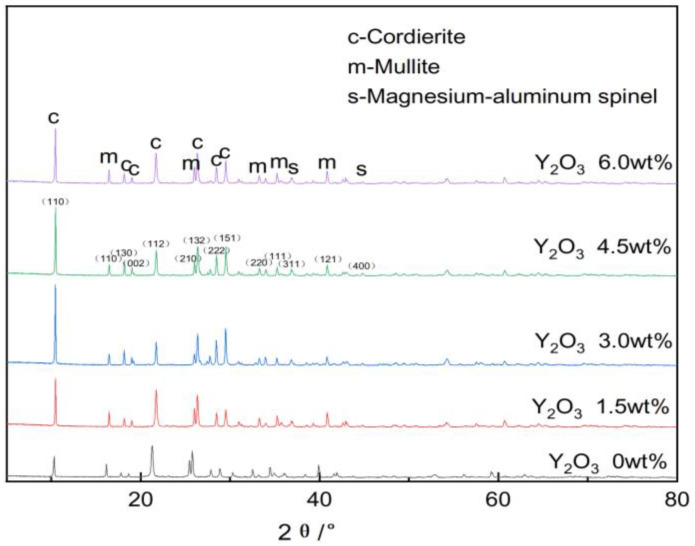
Impact of yttrium oxide addition on the phase composition of the samples.

Figure 6 illustrates the XRD patterns of different samples.

Jade software was utilized to analyze the phase composition of various samples and determine the corresponding Miller indices for each phase, with the results being presented in Figure 6. The analysis reveals that, after sintering at 1350 °C, the primary phases in the sample are cordierite (PDF#13-0294), mullite (PDF#15-0776), and a minor amount of magnesium–aluminum spinel (PDF#21-1152). This indicates that high phase purity can be achieved at a sintering temperature of 1350 °C.

As illustrated in Figure 6, the analysis revealed that, with increasing additions of yttrium oxide, the peak intensity of the cordierite phase initially increased and subsequently decreased. This suggests that the addition of yttrium oxide promoted the formation of cordierite and mullite phases to a certain extent. However, excessive yttrium oxide reduced the peak intensity of the cordierite phase. This effect is ascribed to the ionic radius of Y^3+^ in yttrium oxide being comparable to that of Mg^2+^ in the raw materials, allowing Y^3+^ to partially substitute Mg^2+^, thus causing lattice distortion and the rearrangement of lattice particles. This weakened the bonding force between particles and activated the lattice. Furthermore, the addition of yttrium oxide generated a high-temperature liquid phase within the sample, which moderately heightened the ion migration rate. The solid–gas interface with lower surface tension was replaced by a liquid–gas interface with a higher surface tension, thereby accelerating the solid-phase reactions between ions and promoting the synthesis of the cordierite and mullite phases. However, an excessive liquid phase could partially decompose the cordierite phase, reducing its peak content.

Based on the fundamental physical properties, phase composition, and production cost of the sample, the comprehensive properties of cordierite–mullite composite ceramics clearly satisfy the essential performance requirements stipulated in the YB/T 4549-2016 standard [32] for cordierite–mullite kiln furniture at 1350 °C, with a yttrium oxide content of 3.0 wt%. The primary phase constituents of the sample are cordierite and mullite. Specifically, the incorporation of yttrium oxide not only lowers the synthesis temperature of cordierite–mullite composite ceramics to some extent but also significantly enhances their mechanical properties and high-temperature service performance.

### 3.5. Microstructure

**Figure 7:**
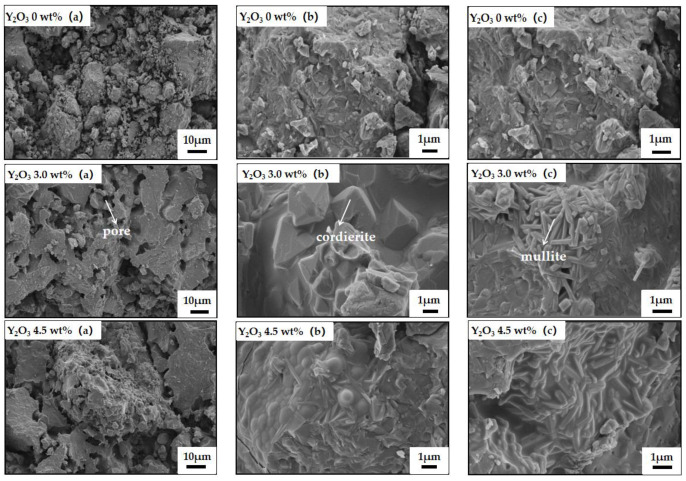
SEM photos of samples with different amounts of yttrium oxide.

Figure 7 presents the cross-sectional microstructures of samples without, with 3.0 wt% and 4.5 wt% yttrium oxide. The microstructure images of the samples are labeled as (a), (b), and (c) respectively. Image (a) is presented at a magnification of 1000×, while images (b) and (c) are shown at 10,000×.

**Figure 8:**
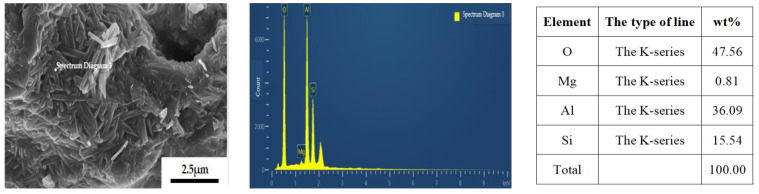
Energy spectrum analysis of the sample containing 3.0 wt% yttrium oxide.

Figure 8 presents the outcomes of the energy spectrum analysis conducted on the sample with a yttrium oxide concentration of 3.0 wt%. An EDS analysis of the sample is presented on page 5, specifically at lines 190 and 195.

Based on the energy spectrum analysis results presented in Figure 8, it is evident that, at a sintering temperature of 1350 °C, the introduction of a specific quantity of yttrium oxide as a sintering additive leads to the formation of a columnar phase with a significantly larger growth diameter. The compositional analysis via energy spectrum indicates that the composition of this columnar phase (O: 48.83 wt%, Al: 38.03 wt%, Si: 13.14 wt%) closely aligns with the theoretical composition of mullite, thereby confirming that the elongated columnar phase formed in the sample is indeed the mullite phase.

As shown in Figure 7, the sample sintered at 1350 °C without a sintering aid displayed a loose structure with poor interparticle bonding. Notably, well-crystallized long-rod mullite phases are absent from the matrix, leading to diminished mechanical properties and reduced thermal shock resistance. In contrast, the sample with 3.0 wt% yttrium oxide presented a dense structure with ribbed plate-shaped particle bonding and evenly distributed micron-sized pores. Furthermore, elongated rod-like mullite grains with an interlocking structure are formed within the cordierite matrix, and well-crystallized short-columnar cordierite grains are observed [33]. The cross-arranged mullite phases not only enhance the mechanical properties of the sample but also mitigate thermal stress during thermal shock. This establishes a critical foundation for advancing the low-temperature synthesis and high-temperature performance of cordierite–mullite composite ceramics. However, the sample prepared with 4.5 wt% yttrium oxide displayed a large amount of liquid phase between the grains, with the pores being filled with a glass phase, markedly increasing the density. This observation is consistent with the aforementioned high bulk density of the sample. Meanwhile, the excessive glass phase was detrimental to the release of thermal stress during rapid cooling/heating, which aligns with the observed decrease in the strength retention rate of the sample.

Therefore, while yttrium oxide as a sintering agent can promote the low-temperature synthesis process of cordierite–mullite composite ceramics to some extent, as well as enhance its mechanical properties and high-temperature performance, the dosage should be carefully controlled. Excessive amounts may have adverse effects.

## 4. Conclusions

(1) The use of yttrium oxide as a sintering aid in the preparation of cordierite–mullite composite ceramics resulted in partial substitution of Mg^2+^ by Y^3+^, forming a limited solid solution and introducing lattice defects. Moreover, the addition of yttrium oxide lowered the formation temperature of the liquid phase and accelerated the diffusion of Al^3+^ and Mg^2+^ to the Si^4+^ surface. This promoted the solid-state reaction, grain crystallization, and densification during sintering. Consequently, the composite ceramics exhibited improved mechanical properties and thermal shock stability, showing the potential for practical applications.

(2) At a sintering temperature of 1350 °C for 2 h, the addition of an optimal amount of yttrium oxide (3.0 wt%) significantly enhanced the phase synthesis process of the composite ceramics. A microstructural analysis indicated that the liquid phase and lattice distortion promoted densification and sintering, stabilizing the structure. The formation of a long rod-like mullite phase with interlocking structures not only increases the resistance to crack propagation but also mitigates the stress concentration during crack propagation, thereby improving the mechanical properties and thermal shock resistance. Specifically, the compressive strength increased by 121%, and the flexural strength increased by 177%. After three thermal shock cycles at 1100 °C, the retention rates of the compressive and flexural strengths were 87.66% and 71.01%, respectively. However, excessive addition of yttrium oxide leads to partial decomposition of cordierite and excessive liquid phase filling in the grain boundaries, which increases density but compromises microstructural stability and reduces high-temperature performance. These findings underscore the critical role of yttrium oxide content in determining the phase composition and structural stability of cordierite–mullite multiphase ceramics.

## Figures and Tables

**Table 1:** Chemical composition of the raw materials (wt%).

Raw Materials	SiO_2_	Al_2_O_3_	Fe_2_O_3_	TiO_2_	MgO	CaO	K_2_O	Na_2_O	ZrO_2_	P_2_O_5_	Other
Calcined kaolin	52.48	44.32	0.46	2.07	0	0.22	0.10	0.12	0.09	0.05	0.09
Sintered magnesia	0.43	0	0	0	97.35	1.24	0	0	0	0	0.98
Alumina powder	99.90	0	0	0	0	0	0	0	0	0	0.10

**Table 2:** Formulation design (wt%).

Sample Designation	Calcined Kaolin	Sintered Magnesia	Alumina Powder	Yttrium Oxide (Sintering Aid)	PVA Solution (Binder)
C-M-Y0	82.18	9.57	8.26	0	3
C-M-Y1	82.18	9.57	8.26	1.5	3
C-M-Y2	82.18	9.57	8.26	3.0	3
C-M-Y3	82.18	9.57	8.26	4.5	3
C-M-Y4	82.18	9.57	8.26	6.0	3

**Table 3:** Linear change upon re-sintering of the samples with different yttrium oxide addition amounts.

Content of Y_2_O_3_/wt%	0	1.5	3.0	4.5	6.0
Before reheating φ_0_(mm)	29.08	28.45	27.87	27.10	26.66
After reheating φ_1_(mm)	29.02	28.41	27.84	27.08	26.64
△φ = φ_1_ − φ_0_(mm)	−0.06	−0.04	−0.03	−0.02	−0.02
LCOR = △φ/φ_0_(%)	−0.2063	−0.1406	−0.1076	−0.0738	−0.0750

Note: A “−” sign indicates dimensional shrinkage of the sample following reheatng.

## Data Availability

The original contributions presented in this study are included in the article. Further inquiries can be directed to the corresponding author.

## References

[1] Zhang Q., Niu J., Zhao Z., Wang Q. (2022). Research on the effect of thermal runaway gas components and explosion limits of lithium-ion batteries under different charge states. J. Energy Storage.

[2] Zhang J., Huang H., Zhang G., Dai Z., Wen Y., Jiang L. (2024). Cycle life studies of lithium-ion power batteries for electric vehicles: A Review. J. Energy Storage.

[3] Shen H., Wang Q., Chen Z., Rong C., Chao D. (2023). Application and Development of Silicon Anode Binders for Lithium-Ion Batteries. Materials.

[4] He S., Wei C., Kang X., Li S. (2023). Effects of Spodumene Addition on the Properties of Saggars for Calcined Lithium Cobalt Oxide Cathode Materials. Mater. Rev..

[5] Sun Z., Yu J., Zhao H., Sang S., Zhang H., Zhang Y., He H. (2022). Damage mechanism and design optimization of mullite-cordierite saggar used as the sintering cathode material in Li-ion batteries. J. Eur. Ceram. Soc..

[6] Phan T., Kim I.T. (2024). Recent Advances in Sodium-Ion Batteries: Cathode Materials. Materials.

[7] Xiang K., Li S., Li Y., Wang H., Xiang R. (2022). Interactions of Li_2_O volatilized from ternary lithium-ion battery cathode materials with mullite saggar materials during calcination. Ceram. Int..

[8] Tuan K., Phong D.H., Lam M.T., Nguyen V.T., Le M.-V., Luan V.H. (2021). A study of cordierite ceramics synthesis from domestic kaolin. Earth Environ. Sci..

[9] Long M., Li Y., Qin H., Xue W., Jiang P., Sun J., Kumar R.V. (2017). Mechanism of active and passive oxidation of reaction-bonded Si_3_N_4_-SiC refractories. Ceram. Int..

[10] Yuan L., Ma B., Zhu Q., Zhang X., Zhang H., Yu J. (2017). Preparation and properties of mullite-bonded porous fibrous mullite ceramics by an epoxy resin gel-casting process. Ceram. Int..

[11] Xiang K., Li S., Li Y., Wang H., Xiang R., He X. (2023). Firing properties and corrosion resistance of mullite-Al_2_TiO_5_ saggar materials. Int. J. Appl. Ceram. Tec..

[12] Duan X., Zheng H., Chen Y., Qian F., Liu G., Wang X., Si Y. (2020). Study on the corrosion resistance of cordierite-mullite and SiC refractories to Li-ion ternary cathode materials. Ceram. Int..

[13] Wu J.F., Yin Y.Q., Fan J., Xu X.H., Shen Y.Q., Yu J.Q. (2024). Preparation, microstructure and properties of cordierite-mullite-corundum porous ceramics for high-temperature gas-solid separation. Ceram. Int..

[14] Wu J., Lu C., Xu X., Zhang Y. (2019). Preparation of Cordierite-mullite Ceramics for Solar Thermal Storage. J. Wuhan Univ. Technol. Mater. Sci. Ed..

[15] Biryukova A.A., Dzhienalyev T.D., Boronina A.V., Khabas T.A., Pogrebenkov V.M. (2017). Effect of Modifying Additions on Synthesis and Properties of Cordierite-Mullite Ceramic from Kazakhstan Resources. Refract. Ind. Ceram..

[16] Sun Z., Yu J., Zhao H., Sang S., Zhang H. (2022). Effects of partial substitution of calcium alumino-titanate on the properties and microstructure of mullite-cordierite composites. Ceram. Int..

[17] Hu P., Zhang J., Li S., Li Z., Li H. (2018). Preparation of cordierite-mullite composites based on high alumina coal ash. Clean Coal Technol..

[18] Xie H.J., Ren Y., Xiao G.Q., Ding D. (2020). Preparation and erosion mechanism of Mullite cordierite sagger. J. Chin. Ceram. Soc..

[19] Kakroudi M.G., Vafa N.P., Asl M.S., Shokohimehr M. (2020). Effects of SiC content on thermal shock behavior and elastic modulus of cordierite-mullite composites. Ceram. Int..

[20] Wang L., Ma B., Ren X., Yu C., Deng C., Liu C., Hu C. (2022). ZrO_2_ and M_x_O_y_ (M = La, Ce, and Nb) synergistically reinforced porous cordierite ceramics synthesized via a facile solid-state reaction. Ceram. Int..

[21] Xu X., Xu X., Wu J., Lao X., Zhang Y., Li K. (2016). Effect of Sm_2_O_3_ on microstructure, thermal shock resistance and thermal conductivity of cordierite-mullite-corundum composite ceramics for solar heat transmission pipeline. Ceram. Int..

[22] Hu C., Xiang W., Chen P., Li Q., Xiang R., Zhou L. (2022). Influence of Y_2_O_3_ on densification, flexural strength and heat shock resistance of cordierite-based composite ceramics. Ceram. Int..

[23] Avcioglu C., Artir R. (2024). Waste to shield: Tailoring cordierite/mullite/zircon composites for radiation protection through controlled sintering and Y_2_O_3_ addition. Nucl. Eng. Technol..

[24] Chen Z.Y. (2005). Chemical Thermodynamics and Refractory Material.

[25] (2015). Standardized Testing Procedures for Determining Bulk Density, Apparent Porosity, and True Porosity of Compacted Shaped Refractory Products.

[26] (2008). Standard Test Method for Determining the Compressive Strength of Refractories at Ambient Temperature.

[27] (1999). Standardized Test Method for Evaluating the Bending Strength of Ceramic Materials..

[28] (2022). Standardized Testing Methodology for Evaluating Permanent Linear Changes in Refractory Materials During Heating Processes.

[29] Donald R., Askeland P.P. (2005). Phule. Essentials of Materials Science and Engineering (Photographic Printing Plate).

[30] Kingery W.D. (1959). Densification during sintering in the presence of a liquid phase. Appl. Phys..

[31] Wu T., Zhou J., Wu B., Xiong Y. (2016). Effect of Y_2_O_3_ additives on the wet abrasion resistance of an alumina-based grinding medium. Wear.

[32] (2016). Cordierite-mullite kiln set..

[33] Qin M., Wang X., Wang Z., Liu H., Ma Y. (2016). Microstructure and Properties of Solid State Synthesized Cordierite with Different Raw Materials. Mater. Mech. Eng..

